# A Case of MRI-Negative Leptomeningeal Disease From Non-small Cell Lung Cancer

**DOI:** 10.7759/cureus.56727

**Published:** 2024-03-22

**Authors:** Andrew Kokavec, Joanna Laba

**Affiliations:** 1 Otolaryngology - Head and Neck Surgery, London Health Sciences Centre, London, CAN; 2 Radiation Oncology, London Health Sciences Centre, London, CAN

**Keywords:** brain metastases, csf sampling, neurological symptoms, radiotherapy (rt), metastatic non-small cell lung cancer, mri, leptomeningeal disease

## Abstract

Leptomeningeal disease (LMD) is a rare complication of advanced non-small cell lung cancer (NSCLC), associated with a poor prognosis. We report the case of a 55-year-old man, who presented with a metastatic NSCLC with limited brain and abdominal metastases. He was treated with both chemoimmunotherapy and stereotactic radiotherapy (SRT) to the brain. Despite treatment, the patient experienced progressive neurological symptoms not in keeping with the extent of disease seen on imaging of the brain. Due to this incongruence between symptoms and radiologic findings, he underwent a lumbar puncture, which had positive cytology for LMD. He had a rapid progression of symptoms and died six days after the discovery of LMD. We review the available literature regarding the prevalence of MRI-negative LMD from a solid primary malignancy.

## Introduction

Leptomeningeal disease (LMD) is a rare pattern of disease progression in patients with metastatic non-small cell lung cancer (NSCLC). Patients may be asymptomatic or have non-specific neurological deficits, making diagnosis difficult [1]. Although still rare, LMD is becoming increasingly common in NSCLC patients due to the increasing length of survival, largely resulting from improved systemic therapies [2].

Diagnosis is primarily made using gadolinium-enhanced MRI, which typically involves imaging the entire brain and spinal cord [3]. LMD presents as an enhancement of the brain surface, sulci, nerves, and nerve roots [3]. Presentations can include nodular, linear, or curvilinear morphology, with both focal and diffuse presentations [4]. MRI sensitivity has been reported as 70-90% in LMD with solid primaries; however, a negative MRI does not exclude LMD [4,5].

The gold standard for the diagnosis of LMD is serial cerebrospinal fluid (CSF) sampling, looking for malignant cells [3]. Other CSF findings in LMD include pleocytosis, hypoglycorrhachia, and elevated protein, along with a raised opening pressure above 200 mmHg [1]. Sensitivity begins at 50% with the initial puncture, increasing to 80% with the second sample, with each subsequent puncture raising the sensitivity by 2-5% [3].

Although treatment intent is palliative, a timely diagnosis is vital to control symptoms and preserve the quality of life [1]. Treatment typically involves radiotherapy delivered to the whole brain or focal spinal regions. Chemotherapies may also be used, but evidence supporting their efficacy is limited as they may not adequately penetrate the blood-brain barrier [6].

We report on a case of LMD that initially presented with limited brain metastases treated with stereotactic radiotherapy (SRT). Soon after, the patient developed non-specific neurological symptoms including nausea, vomiting, syncope, and photophobia. Despite having an NSCLC primary, serial MRIs, including four MRI heads and one MRI spine, showed no evidence of LMD. It was only after a lumbar puncture and CSF sampling that LMD was diagnosed. The patient had a rapid progression of neurological symptoms and died six days after the diagnosis of LMD by lumbar puncture.

## Case presentation

Initial presentation and diagnosis

**Figure 1:**
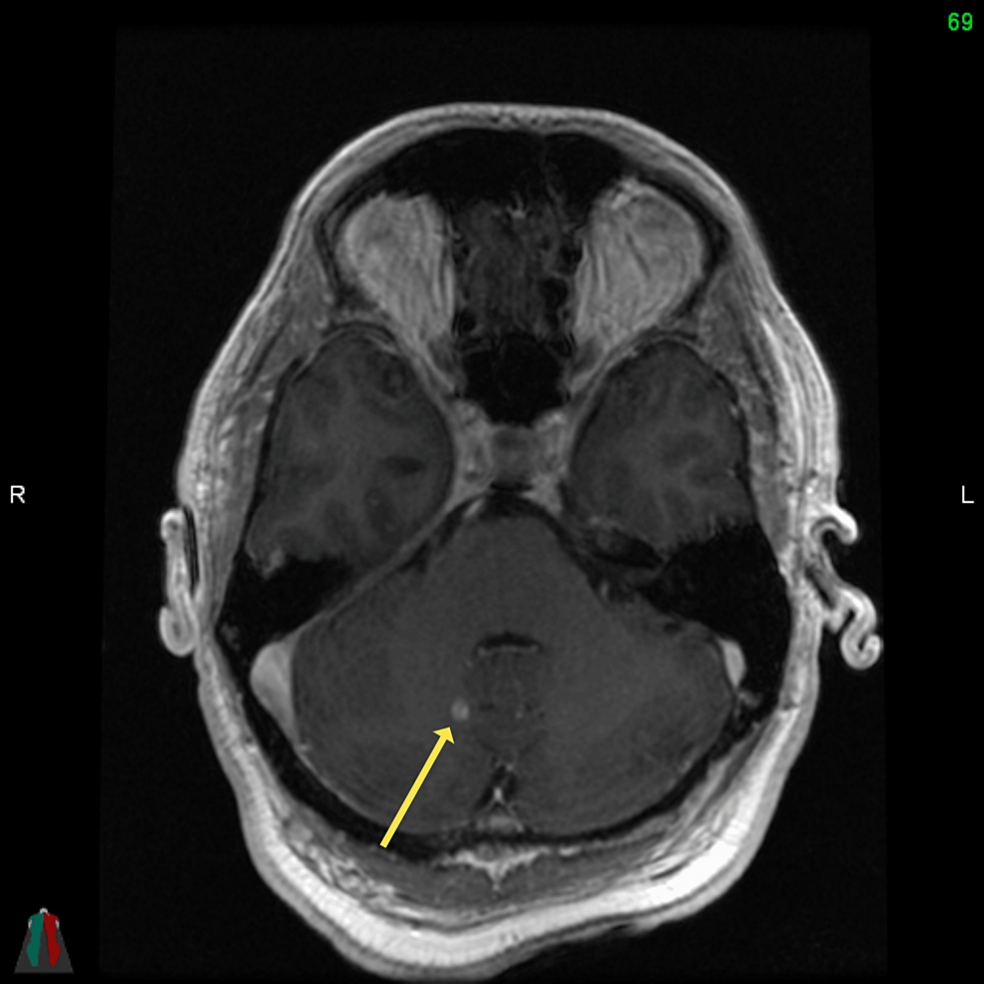
Axial head MRI T1 FSPGR post-contrast demonstrating right cerebellar hemisphere nodule measuring 5 mm in keeping with metastatic deposit FSPGR: fast spoiled gradient-echo

**Figure 2:**
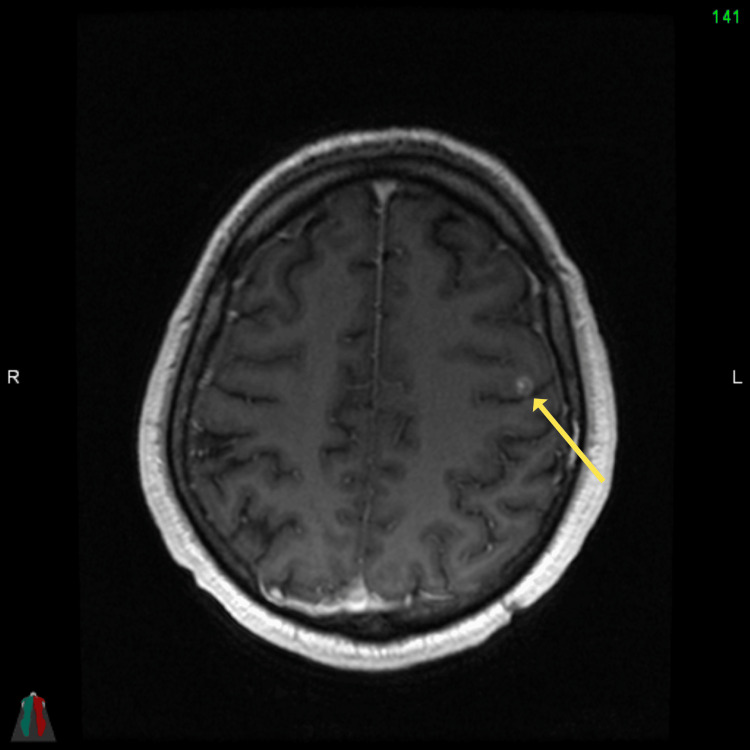
Axial head MRI T1 FSPGR post-contrast demonstrating left frontal lobe nodule measuring 6 mm in keeping with metastatic deposit FSPGR: fast spoiled gradient-echo

A previously healthy male and lifetime non-smoker presented to the emergency department with a nine-month history of cough associated with clear sputum production. The patient also had a 12-month history of weight loss and a three-month history of night sweats. CT imaging demonstrated a left anterior mediastinal mass with pleural and pericardial invasion. Additionally, there were metastatic mediastinal and right hilar lymphadenopathy and a large malignant pleural effusion, findings that were consistent with stage 4 lung cancer. An endobronchial ultrasound (EBUS) biopsy was conducted, which demonstrated an adenocarcinoma of the lung that was PD-L1 weakly positive and ALK, ROS1, and EGFR negative. The patient was initially treated with chemoimmunotherapy, including carboplatin, pemetrexed, and pembrolizumab. Five months later, repeat CT and MRI (fine slice, T1-weighted post-gadolinium) showed signs of brain and abdominal metastases; see Figure 1 and Figure 2.

Seven brain metastases were treated with stereotactic radiation to a dose of 20 Gy in 1 fraction. The patient's chemotherapy treatment regimen was then changed to carboplatin plus gemcitabine. He was also started on dexamethasone 4 mg daily.

Subsequent presentation

Fifty-three days after completing stereotactic brain radiation, the patient began to experience worsening neurological symptoms that prompted presentation to the emergency department. These included severe occipital headaches with radiation to the frontal region bilaterally. They were also positional in nature, worsened by lying down and improved by standing. Additionally, the patient had binocular diplopia, nausea, vomiting, and orthostatic dizziness. He also experienced two episodes of syncope.

Physical examination revealed the following vitals: temperature, 36.5; heart rate, 46; respiratory rate, 14; blood pressure, 137/87; oxygen saturation, 100% on 3L nasal prongs; and Glasgow Coma Scale (GCS): 15.

On examination, there were no papilledema and no venous pulsations. Extraocular movements were intact, with full visual fields. Pupils were equal, round, and reactive to light and accommodation. There was no facial asymmetry. The hearing was intact bilaterally. The tongue and palate were midline. Sensation in the face and extremities was intact. There was no pronator drift. Strength was 5/5 symmetrically over the upper and lower extremities.

**Figure 3:**
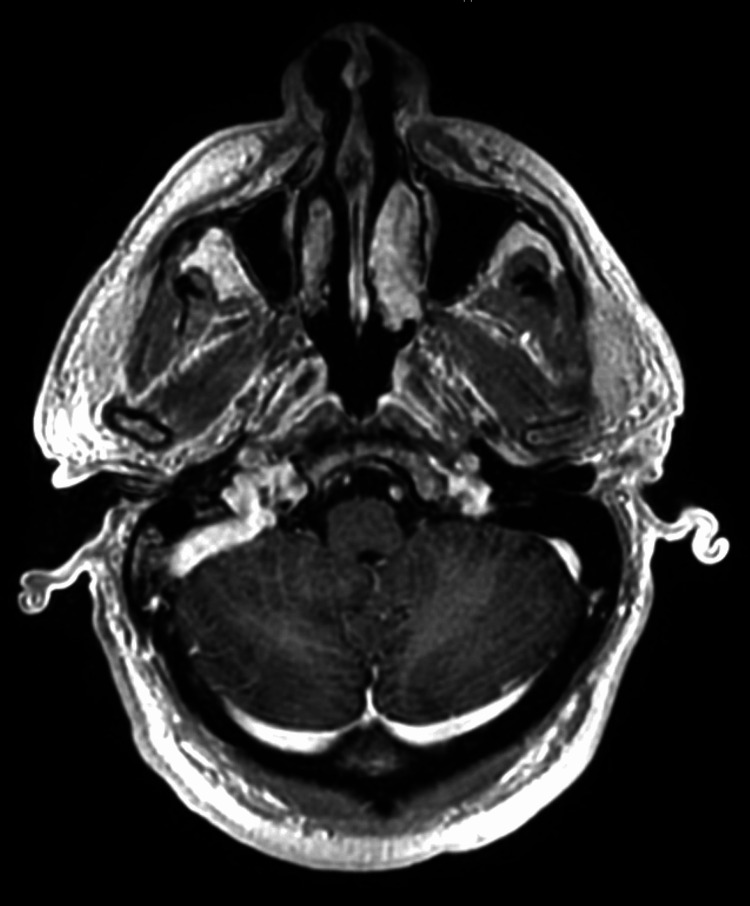
Axial head MRI T1 post-contrast at the level of the cerebellar folia showing no abnormal enhancement or signs of leptomeningeal disease

**Figure 4:**
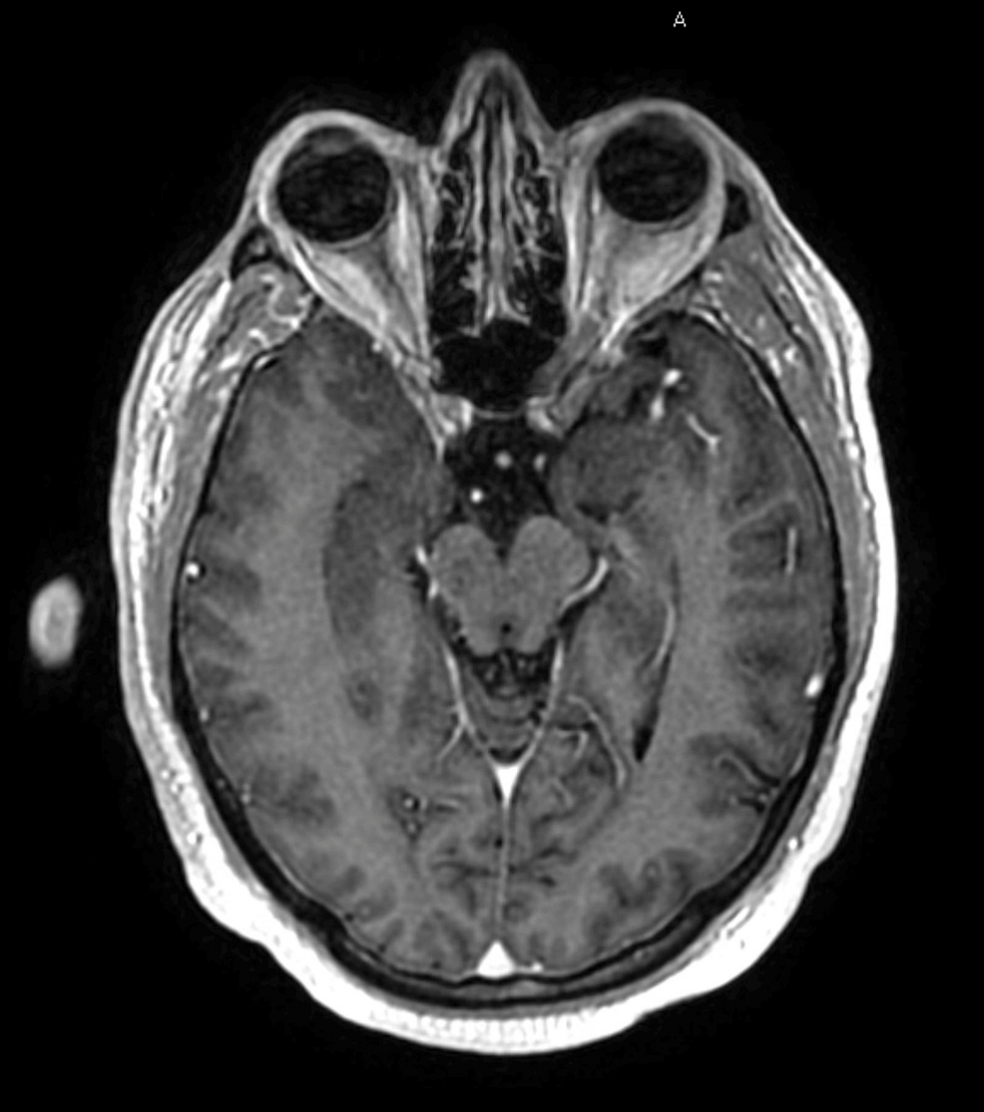
Axial head MRI T1 post-contrast at the level of the optic nerves showing no abnormal enhancement or signs of leptomeningeal disease

**Figure 5:**
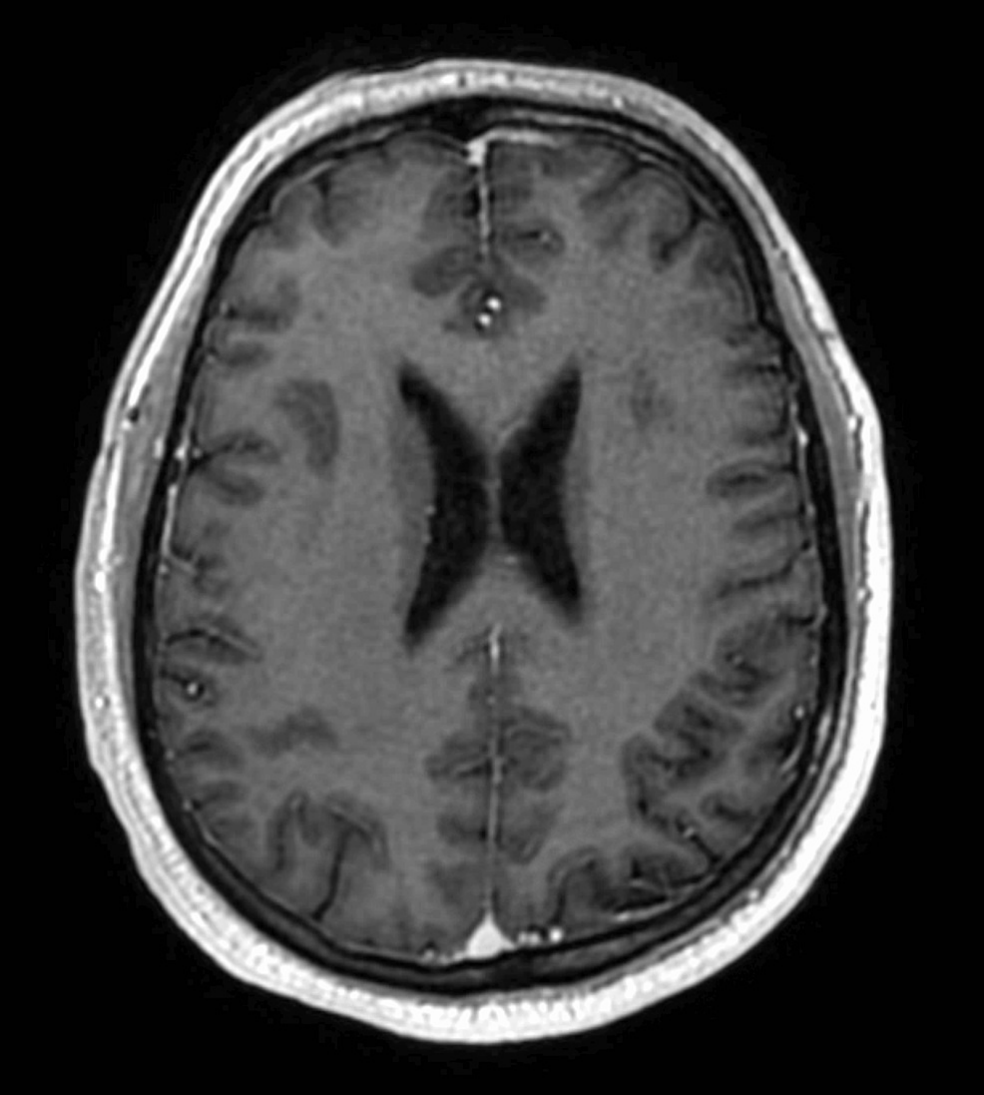
Axial head MRI T1 post-contrast at the level of the lateral ventricles showing no abnormal enhancement or signs of leptomeningeal disease

Repeat MRI did not demonstrate worsening of his intracranial lesions, edema, or other clear causes for his symptoms; see Figure 3, Figure 4, and Figure 5.

**Figure 6:**
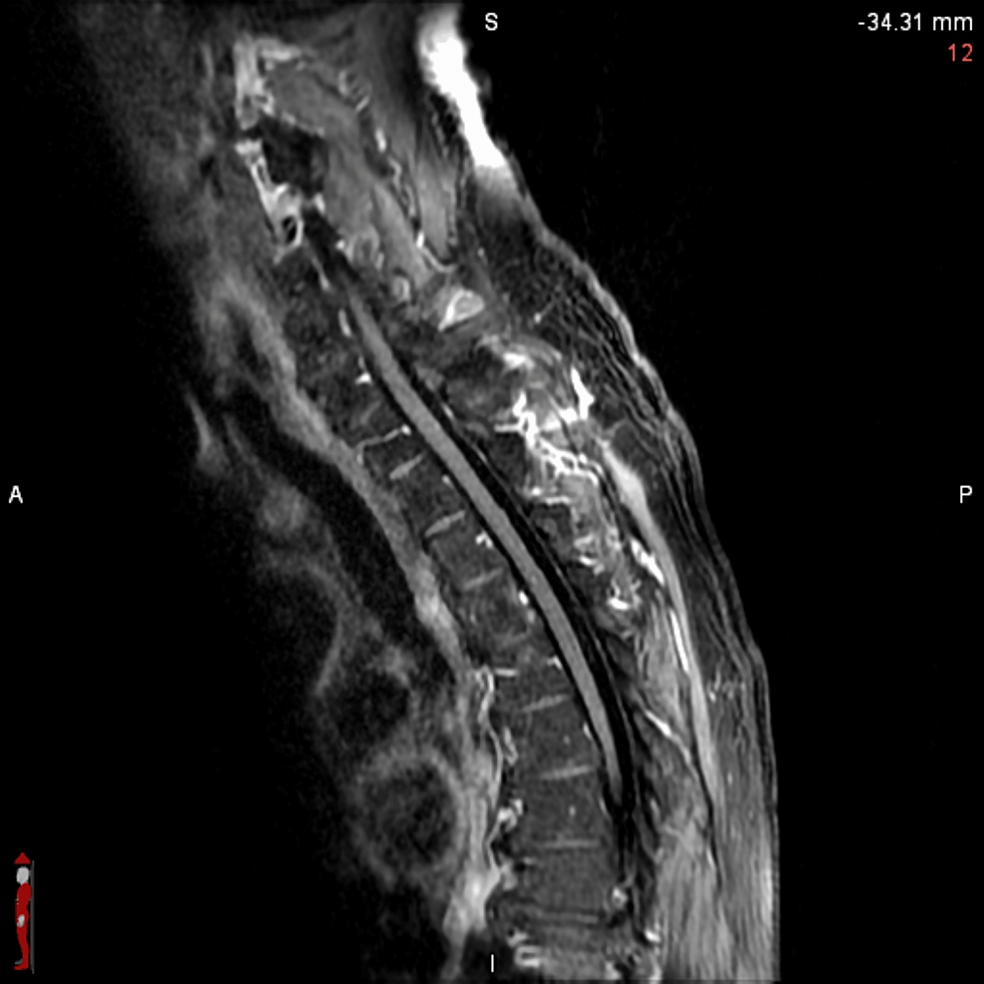
Sagittal spine MRI T1 FLAIR-FS from C2 to T9 showing no abnormal enhancement or signs of leptomeningeal disease FLAIR-FS: fluid-attenuated inversion recovery with fat suppression

**Figure 7:**
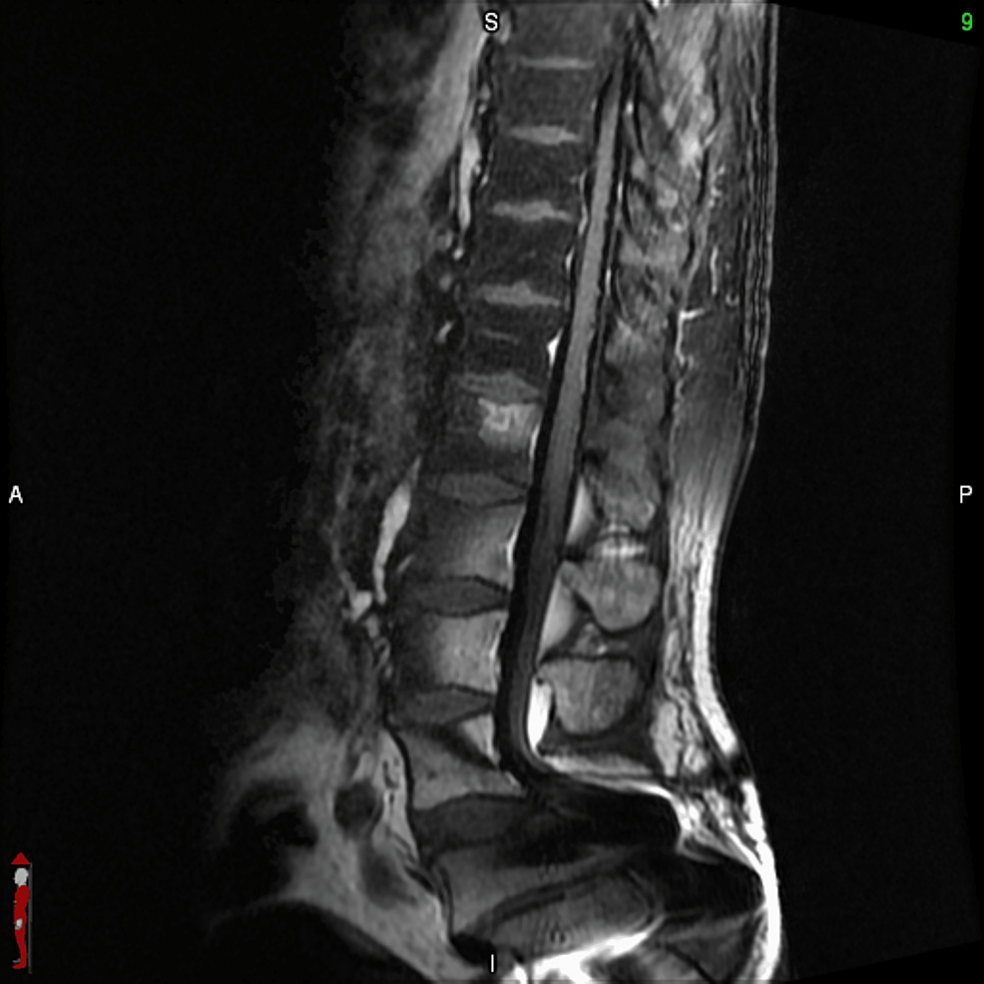
Sagittal spine MRI T1 FLAIR-FS from T8 to S1 showing no abnormal enhancement or signs of leptomeningeal disease (image degradation caudal to the level of L4-5 onwards due to metallic rods and screws) FLAIR-FS: fluid-attenuated inversion recovery with fat suppression

The neurology service was consulted and felt the headaches were being caused by increased ICP without an exact cause. The patient was started on acetazolamide for the treatment of possible idiopathic intracranial hypertension. His dexamethasone dose was increased to 4 mg twice daily. A further MRI was conducted to rule out sinus venous thrombosis, which was negative. An MRI of the spine was also conducted to rule out leptomeningeal metastases (see Figure 6 and Figure 7), which was also negative. 

Without a clear origin for the patient's headaches, a lumbar puncture was performed to assess intracranial pressure (ICP) and obtain CSF for analysis. The opening pressure was over 55 cm H2O despite no evidence of hydrocephalus on CT. Four tubes of CSF were collected and sent for analysis. This demonstrated evidence of malignant cells on cytology, confirming the diagnosis of LMD, despite several MRIs showing no radiologic evidence of leptomeningeal deposits; see Table 1. A CT of the head was completed after the lumbar puncture, which confirmed no cerebellar tonsillar herniation/ectopia.

**Table 1:** CSF analysis results CSF: cerebrospinal fluid; H: high

Results	First collection	Second therapeutic collection (three days after the first)
Appearance after centrifugation	Clear and colourless	Bloody/yellow and clear with a red cell button
Glucose, CSF	2.9	H 4.4
Protein, CSF	H 870	H 692
Nucleated cells, CSF	H 13	H 17
Erythrocyte, CSF	6	7,000
Neutrophil %, CSF	2.0	27.0
Lymphocyte %, CSF	12.0	22.0
Monocytoid %, CSF	86.0	51.0
Cytology/flow cytometry	Moderately cellular sample. Malignant cells present singly and in a few clusters. The cells are large with eccentric nuclei, irregular nuclear contours, prominent macronucleoli, and vacuolated cytoplasm. The background consists of debris and occasional lymphocytes. Flow cytometry CSF: The lymphocyte population represents approximately 18% of the total leukocyte count and consists of T cells expressing either helper or suppressor immunophenotype. Too few B cells to assess light-chain expression. Overall features are that of an adenocarcinoma consistent with lung primary given the history	N/A

The patient's symptoms progressed rapidly, and given his poor performance status, all active treatments were stopped, and his symptoms were managed with comfort measures by the palliative care team. His dexamethasone dose was also further increased to 8 mg twice daily. Therapeutic lumbar puncture was completed three days after the first diagnostic collection to treat ongoing severe headaches. The opening pressure was 22 cm H2O. The patient died six days after the diagnosis of LMD.

## Discussion

This patient with metastatic adenocarcinoma of the lung began to experience non-specific neurological findings after stereotactic radiotherapy for limited brain metastases. These included positional headaches, binocular diplopia, nausea, vomiting, and orthostatic dizziness in keeping with an increased ICP. Full neuroimaging including a gadolinium-enhanced MRI was performed. Serial MRIs did not identify an underlying cause to explain his symptoms. It was not until the CSF was sampled that the diagnosis of LMD was made.

Diagnosing LMD

Upon review of the literature, gadolinium-enhanced MRI continues to be considered the mainstay for diagnosing LMD [6]. This presents as an enhancement of the brain's surface and nerves, with both focal and diffuse presentations [7,8]. Sensitivity has been reported to be 70-90% in LMD with solid primaries [4,5]. Despite this, previous studies have demonstrated a wide variation in reporting LMD neuroimaging, with poor inter-observer agreement between radiation oncologists and neuro-radiologists [9]. Therefore, the gold standard remains serial CSF sampling, which initially has a low sensitivity of 50-60% but rises with each subsequent puncture [10]. CSF can be analyzed for malignant cells and protein levels, as well as high opening pressure. Despite this, 10% of patients can have a normal CSF profile, meaning the diagnosis of LMD can be missed [2]. CSF flow studies are also a helpful adjunct as 30-70% of patients with LMD will have obstructed flow [3].

However, a lumbar puncture is an invasive test and comes with associated risks including bleeding, infection, or cerebral herniation. This procedure may not be appropriate for patients at the end of life. 

The sensitivities of these diagnostic modalities are low on their own; therefore, it is quite possible to miss the diagnosis when any of these are used independently. This can make the diagnosis of LMD challenging, and therefore, it is imperative that clinicians use CNS imaging and CSF analysis in tandem and ensure that these correlate with the clinical presentation. Further complicating diagnosis, recent surgery, infection, or radiation can cause abnormal leptomeningeal enhancement, often resembling LMD [6]. Diagnostic algorithms have been proposed by Wang et al. that include signs and symptoms, MRI of the brain and spine, and serial CSF cytology ± flow cytometry in a step-wise approach to diagnose LMD [10]. This underscores the necessity of keeping LMD on the differential diagnosis, regardless of imaging findings.

The incidence of LMD continues to increase as the survival of cancer patients is being extended with novel treatments [6]. Despite this, many anti-neoplastic agents have poor CNS penetration and therefore limit efficacy behind the blood-brain barrier. It is estimated that intracranial metastases accompany LMD in 98% of patients with non-leukemic primary cancer [11].

Patients with brain metastases are increasingly receiving treatment with stereotactic radiotherapy rather than whole-brain radiation. A retrospective review conducted by Trifiletti et al. concluded that approximately 14% of patients with brain metastasis from breast cancer developed LMD following stereotactic radiosurgery (SRS) [12]. Therefore, it is important for clinicians to remain vigilant and consider LMD in the differential diagnosis, especially when a patient's neurological status changes.

Despite early diagnosis, the prognosis for those with LMD remains poor. The time from diagnosis to death is approximately four to six weeks if left untreated [3]. With treatment, average survival increases to two to four months, with breast cancer patients having the best prognosis [3]. Patients with an NSCLC have a median survival of 3.5 months with treatment, with improved survival in those who are EGFR positive and receiving treatment with tyrosine kinase inhibitors [3]. Therefore, it is important that LMD is diagnosed promptly to improve patient survival and reduce symptoms. 

## Conclusions

Diagnosing LMD remains a challenge despite advances in non-invasive imaging. The low sensitivity of initial CSF sampling further contributes to the difficulty in diagnosis. 

The patient discussed herein began to experience non-specific neurological symptoms including headaches, diplopia, and nausea after treatment with SRT for limited brain metastases. While he did have brain metastasis, their size and characteristics were not sufficient to explain these symptoms. Despite having multiple MRIs, none were able to diagnose his LMD. The diagnosis was not made until CSF was sampled, several weeks after the onset of his symptoms.

Our patient demonstrates the importance of keeping LMD on the differential diagnosis in patients with malignancies, even in those with reassuring MRIs. LMD is becoming an increasingly prevalent complication of malignancy, and the prognosis remains quite poor. However, earlier diagnosis and treatment can improve symptom management and survival.
